# A modular optical honeycomb breadboard realized with 3D-printable building bricks and industrial aluminum extrusions

**DOI:** 10.1016/j.ohx.2021.e00182

**Published:** 2021-03-05

**Authors:** Yannic Toschke, Bjoern Bourdon, Dirk Berben, Mirco Imlau

**Affiliations:** a Department of Physics, Osnabrueck University, Barbarastrasse 7, Osnabrueck D-49076, Germany; b Fachhochschule Suedwestfalen, Haldener Strasse 182, D-58636 Iserlohn, Germany

**Keywords:** Optical breadboard, Photonics, Optics, 3D-printing, Industrial aluminum extrusions, Agile prototyping, Interferometer, Spectrograph

## Abstract

Optical breadboards with honeycomb structure provide a solid surface with mounting hole grids for building optical assemblies, sub-systems and experiments in the fields of quantum-optics and photonics. Performance criteria are the ability to resist bending under load (stiffness) and the ability to dissipate induced vibrations to the board (damping). The hardware presented in this paper deals with the possibility of assembling optical breadboards using 3D-printed building bricks with honeycomb structure, so-called ’breadboard bricks’, and industrial aluminum extrusions, so-called ’breadboard profiles’. With this do-it-yourself approach, it is possible to make changes to the breadboard, such as making an opening, changing its shape or increasing the mounting surface whenever needed. Furthermore, the breadboard is automatically compatible with industrially relevant mechanical design platforms. Aluminum extrusions and the PLA thermoplastic filament provide mechanical stiffness and damping, respectively. Further characteristics are low costs and a modular design. All this makes it especially suited for agile prototyping of (laser) optical assemblies in many engineering processes.

## Graphical abstract

**Figure:**
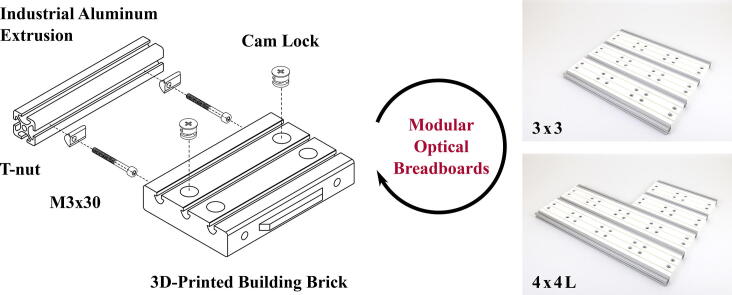

## 1 Hardware in context

Optical breadboards are the backbone for high-precision laser- and optics-related experiments. They permit the secure attachment of optical fixtures and related devices to their top surface. Optical breadboards feature a large stiffness to resist bending under load and the ability to dissipate induced horizontal and vertical vibrations on the board’s surface. Thus, the alignment of optical elements remains stable over time.

**Table:** 

**Specifications table:**		

**Hardware name**	*Optical breadboard***Subject area**	
	• Educational Tools and Open Source Alternatives to Existing Infrastructure	
**Hardware type**		
	• Photonics Tool	
**Open source license**	This work is licensed under the Creative Commons Attribution-ShareAlike 4.0 International License.	
**Cost of hardware**	3 x 3 Breadboard (36 x 32 cm^2^ in size): 91.83 €	
**Source file repository**	https://nbn-resolving.org/urn:nbn:de:gbv:700-202010023619	
	https://nbn-resolving.org/urn:nbn:de:gbv:700-202012143905	

Optical breadboards are generally constructed in a sandwich-like plate structure: Comparatively thin upper and lower metal plates are bonded to a central honeycomb structure, so that a particularly high mechanical stability and a minimum of bending is achieved at the same time as low weight [1]. Structural dampers are mounted in the honeycomb structure to secure vibration damping. The top surface has a plurality of tapped holes for mounting equipment, that are sealed to avoid contamination of the breadboard core [2], [3], [4]. The entire construction is framed with bordering side elements. Some of them are used to increase the board stability against shear forces [5] or for damping treatment [6].

The optical breadboard can be combined with legs containing pneumatic vibration isolators. These act as mechanical low-pass filters reducing the transmission of vibrations from the floor to the tabletop. From a fundamental viewpoint, the system properties can be described with the vibration theory for damped harmonic oscillation of a pendulum (horizontal vibrations) and of a spring (vertical vibrations) [7], [8], [9]. Historically, granite slabs on industrial springs that obey a low resonance frequency in the sub-5-Hz regime have been used as optical support. Thus, the stability of optical setups against a spectrum of acoustic and mechanical frequencies is secured, typically ranging from 10 to 1.000 Hz. While granite tables provide the flatness and rigidity required, their great weight and the difficulty of attaching components to the surfaces make them cumbersome to use. Still, these constructions are preferred for specific setups with extreme requirements to stiffness [10].

Heavy sandboxes, decoupled from the environment by air hoses, were an interesting alternative to granite slabs for many years. These constructions have become widespread, especially in the field of holography [11] and allow the simple positioning of optical components and objects in the sand volume [12]. Because they are easy to assemble/disassemble as well as inexpensive, sandboxes can be seen as the origin of do-it-yourself (DIY) constructions for optical breadboards and tables. Further DIY-approaches combine thin, low-weight granite slabs in sandboxes. But there are also several ‘cheap & easy’-examples that combine a thin plate of aluminum or wood with foam materials or rubber (see e.g. reference [13]). Here, the combination of several stiff materials with different speeds of sound produces a breadboard for which a wide range of vibrations are critically damped, also referred to as composite breadboards, commercially available nowadays [14]. In recent years, 3D-printing technology entered the field of DIY-production of optical baseplates [15], [16], [17]. Also, the combination of 3D-printed optical elements mounted on a solid rail system [18] was proposed, according to the original invention of optical benches of A. Weinhold [19]. Other examples focus on open cage structures [20], [21] according to the LINOS microbench concept [22] that, however, dispenses with the use of optical breadboards.

The first approach of a DIY open source optical breadboard with honeycomb structure was presented and published from the myphotonics.eu project [23]. The basic idea is to use commercially available bricks from ‘LEGO® Systems A/S’ for building complex mechanical and optomechanical structures [24], but without destruction of the bricks. The breadboard stiffness enabled a variety of high-precision laser-experiments from interferometry and holography, but also from microscopy (optical tweezer) and laserphysics (intra-cavity frequency-doubled YAG:Nd laser) [25], [26]. Even a handheld laser power meter was built to determine the breadboard long-term stability by additionally including mini-computer technology (‘Arduino®’, ‘Raspberry Pi®’) [27]. Today, these developments - in particular the ones making use of 3D-printed optomechanics - show a large impact in the field of education.

Despite these numerous DIY approaches, however, there is currently no open source optical breadboard design that is based on the interplay of (i) the tools available today in the field of additive manufacturing, (ii) the abundance of modern material systems and (iii) that takes into account, both the established honeycomb design solutions and hybrid concepts for optical breadboards. In more detail, the literature lacks a professional composite optical breadboard that can be constructed by the user in a modular and scalable way at low costs, that fulfills the requirements for mechanical stability and damping in equal measure and allows the construction of high-precision laser- or optically related experiments without any restrictions. The hardware presented within this work solves this problem and introduces a new class of optical breadboards in the concept of open source hardware [28], [29]. Remarkably this new class features convertibility, scalability and the possibility to disassemble and re-use the breadboard to a high degree. The original approach combines a modular concept, the use of 3D-printing technology, a honeycomb core, nuts and grooves for mounting optical components, a cam lock connection technology and aluminum extrusions that are widely applied in industry. As for the composite breadboards, 3D-printed ‘breadboard brick’ modules with honeycomb structure of PLA thermoplastic filament are combined with industrial aluminum extrusions. This provides damping by the mechanical properties of PLA, while the mechanical stiffness is due to the combination of structures vertical (honeycomb) and horizontal (grooves) to the breadboard surface. In addition, a nut and groove system is used for mounting optical elements following the idea of optical benches. This makes the board suitable for the assembly of optical setups in inline geometry, i.e. setups with optics aligned along a single axis - but is not restricted to this case.

As a result, our optical breadboard is suited for high-precision optical experiments, such as interferometric setups that require stability in the range of fractions of the wavelength over the duration of seconds up to minutes. Due to its low-cost, low-weight, scalability, reusability, ease of integration in industrial environment and professional features, the optical breadboard is of interest for small-to-medium enterprises (SMEs), start-ups, industry and cutting-edge research, but also for private individuals who have little space available or who do not have fixed installation locations.

## 2 Hardware description

The optical breadboard is modularly composed of 3D-printable building blocks, so-called ’breadboard bricks’ (’BBB’), and industrial aluminum extrusions, so-called ’BB profiles’. Exemplarily, we manufactured the bricks by using a 3D-printer (type: I3MK3S) from ‘Prusa Research a.s.’, PLA thermoplastic filament (type: Prusament PLA) from ’Prusa Polymers a.s.’ and aluminum extrusions (type: Profile 5, 20x20, natural) from the company ‘item Industrietechnik GmbH’. However, other 3D-printers, thermoplastic filaments and industrial construction tools can be used as well.

As original approach, unit cells of the same size of 12 x 12 cm^2^ are used to assemble the optical breadboard. Compared to state-of-the-art optical breadboards, benches and tables, the hardware presented here is characterized by its modular and scalable nature, which not only allows the low-cost realization of individual sizes (m x n; m,n ∈N) and geometries (L-shaped, U-shaped, O-shaped,…), but also the possibility to make easy customizations ’on the fly’ in context of mounting and/or adjustment of optical experiments. The design of the ’BBBs’ addresses three essential aspects that are required for a successful implementation of a 3D-printable optical breadboard: (I) A mechanical stable internal structure which secures stiffness and counteracts the potential deformability of the PLA thermoplastic filament. (II) A reliable connection between the ’breadboard bricks’ and the ’BB profiles’ which is easily applicable and provides a stable and rigid surface. (III) Mounting hole rails allowing for a pre-aligned assembly of all kinds of mechanical and opto-mechanical components.

In the context of 3D-printed structures, the attachment of additional components (e.g. mounts for optics) to the printed structure by utilizing screws is of major importance. Typically, threaded holes are realized by the use of nuts, which are inserted into extra pockets in the base structure, or by the insertion of threaded metal sleeves (melt/bond). The alternative approach pursued in this article can be understood as a further development of the first solution. By combining special T-nuts (‘item Industrietechnik GmbH’) together with appropriate grooves in the 3D-printed structure, a variable pre-aligned optics mounting system can be achieved. Remarkably, the grooves are designed such that different thread diameters (M3 or M4) can be used.

The connection technology between the 3D-printed ’BBBs’ and the structure-stabilizing ’BB profiles’ is of fundamental importance. The connection must feature a high mechanical stability, an ease of accessibility as well as a quick & resealable operation. These requirements are met by using a cam lock connection. This type of connector is able to convert an applied torque into a translation rotated by less then 180° which allows for the connection between the ’BBBs’ and aluminum construction profiles to be parallel to the plane of the optical breadboard. It is accessible from the front side. In addition, the maximum force is achieved within half a rotation, which also fulfills the aspect of quick operation.

The use of PLA thermoplastic filament in combination with aluminum offers further advantages: Due to the low kilogram price of PLA (Prusament PLA: 24.99 €/kg) and aluminum extrusions, the presented hardware is considerably cheaper than conventional systems. It also results in a significant weight reduction. Far more important is, that PLA has ideal damping properties due to its elasticity module. Furthermore, we would like to add, that the ubiquitous availability of the aluminum extrusions as engineering and construction tool allows for a direct connection of the optical breadboard with other industrial constructions.

• This hardware allows the user to build optical breadboards of different size and shapes with an emphasis on convertability, remountability and scalability.
• This hardware is compatible with established construction and state-of-the-art building kit systems.
• This hardware opens optical breadboards to the broad public, small-and-medium-sized enterprises (SMEs) and start-ups.
• This hardware allows for an easy and fast adjustment of optical components ‘on the fly’ by combining the individual advantages of 1D optical benches and 2D optical breadboards.
• This hardware is much cheaper than comparable established solutions due to its reusability and the use of 3D-printing technology in combination with industrial aluminum extrusions.

## 3 Design files

**Fig. 1:**
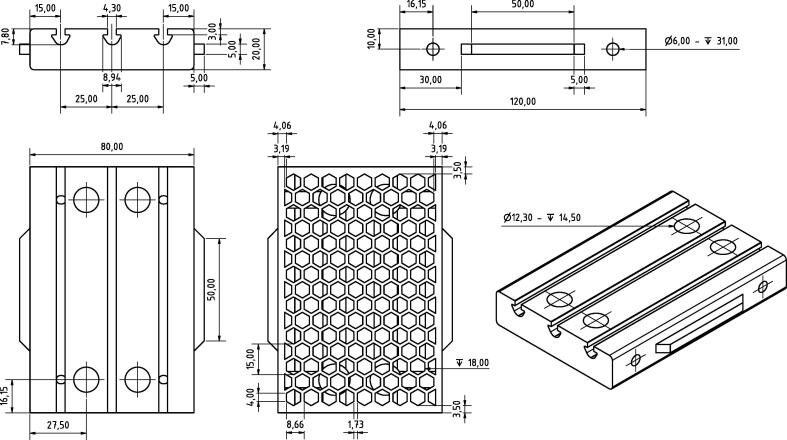
Detailed technical drawing of the ’breadboard brick’. For further information and details use the available ’.stl’ file.

The design file required for 3D-printing the ’breadboard brick’ is directly available with the article in form of a technical drawing (see Fig. 1) and uploaded as a ’.stl’ file to a corresponding repository.

**’breadboard brick’:** Detail drawing used for 3D-printing of the ’breadboard brick’ module.

**Table:** 

**Design filename**	**File type**	**Open source license**	**Location of the file**

’breadboard brick’	detail drawing	Creative Commons Attribution-ShareAlike 4.0 Int. License	available with the article
’breadboard brick’	.stl	Creative Commons Attribution-ShareAlike 4.0 Int. License	File download available at https://nbn-resolving.org/urn:nbn:de:gbv:700-202010023619

## 4 Bill of materials

**Table:** 

**Designator**	**Component**	**Number**	**Cost per unit currency**	**Total cost**	**Source of materials**	**Material type**

BBB	breadboard brick	9	2.30 €	20.70 €	Prusa Polymers a.s.	PLA thermoplastic
BB profile	industrial extrusions 5; 20×20×360 mm^3^, natural	4	5.35 ${€}^{I}$	21.40 €	item Industrietechnik GmbH	aluminum
BB Cam Lock	Cam Lock Rastex 12	36	0.19 €	6.84 €	HORNBACH Baumarkt AG	steel
BB Bolt (M3)	M3×30 Bolts DIN 912	36	0.06 €	2.21 €	Frantos GmbH & Co. KG	stainless steel
BB Nut (M3)	T-Slot Nut 5 St M3	36	0.79 €	28.44 €	item Industrietechnik GmbH	steel
BB Nut (M4)	T-Slot Nut 5 St M4	18	0.68 €	12.24 €	item Industrietechnik GmbH	steel

^I^ The stated costs are made up of the price per meter plus a fixed fee of 2.80 € per cut.

**Fig. 2:**
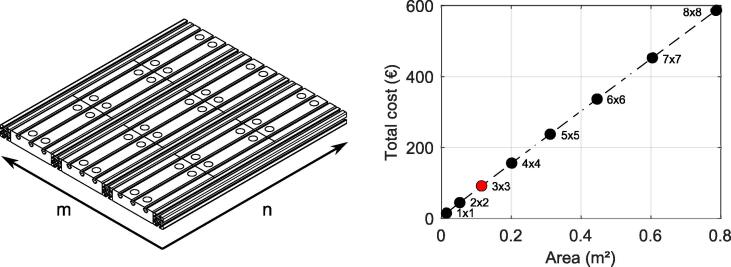
Left: Sketch of the 3 x 3 breadboard highlighting the used nomenclature (m x n; m,n ∈N) of dimensions. Right: Total cost of square sized breadboards (n x n) as a function of resulting area. The cost of a 3 x 3 breadboard is highlighted in red.

We can calculate the costs of a m x n breadboard depicted in Fig. 2 (left) using the formula:

(1) Totalcost(m,n)=m·n·7.82€+(m+1)·(n·0.85€+2.80€)

Here, the given prices are summed-up totals from the bill of materials. As shown in Fig. 2 (right), the costs scale linearly with the breadboard area and we can deduce a price per area of 7.97 €/dm^2^. For comparison, breadboards from most common distributors are offered for 13.33 €/dm^2^ (solid aluminum, 30 x 45 cm^2^) or 45.69 €/dm^2^ (honeycomb, 30 x 45 cm^2^) rendering the presented hardware two to six times cheaper, respectively.

## 5 Build instructions

This chapter provides and focuses on general and detailed step-by-step instructions for the construction of a 3 x 3 optical breadboard suitable for small scale optical experiments. In an additionally provided section, design decisions and possible alternatives are discussed. A supplemental video showing the building concept and the assembly of the 3 x 3 optical breadboard is available through the following URL: https://nbn-resolving.org/urn:nbn:de:gbv:700-202012143905.

### 5.1 General tips

In this short section general tips on the building and 3D-printing aspects are provided. The main focus is on 3D-printing and the required accuracy of fit. In addition, aspects of planning are addressed in advance.

• As a first step the desired size (m x n) of the optical bench should be estimated to calculate the required amount of ’breadboard bricks’. Bear in mind that depending on the printer settings, the printing of one ’BBB’ may take up to 15 hours.
• To improve the 3D-printing speed a lower *z*-resolution (= larger layer thickness) might be useful depending on the desired quality. We note that a lower *z*-resolution may lead to a distortion of overhanging structures. The ’breadboard bricks’ used in this work were printed with a 200 μm resolution.
• It is advised to print the ’breadboard brick’ in a vertical position to eliminate the necessity for additional support structures.
• To prevent the ’breadboard brick’ from falling over while printing the application of an intermediate bonding agent, such as glue stick is adviced. (This tip only holds true for a vertical printing position.)
• As tool for building the breadboard we suggest a ’Philips’ screwdriver size #4.

### 5.2 Preparing the ’breadboard brick’ and the ’breadboard profile’

**Fig. 3:**
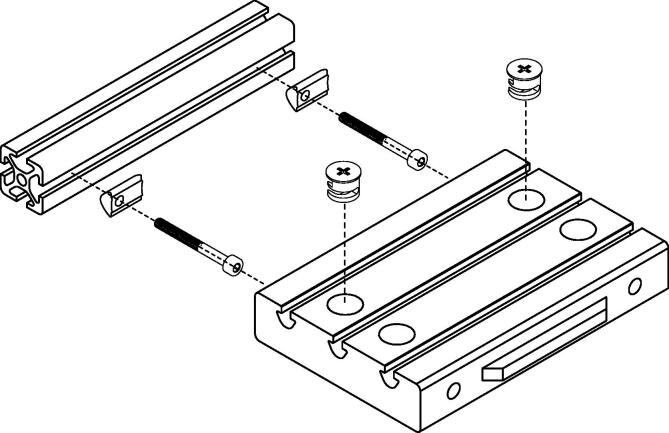
Building concept of the ’breadboard brick’.

This section gives a guideline for the assembly of the ’breadboard brick’ with the ’breadboard profile’. It describes the main rules to get a stable connection which is the prerequisite for the build-up of an optical breadboard (shown in the subsection thereafter). Fig. 3 schematically sketches the connection concept.

(a) Insert the cam locks into corresponding holes in the four corners of each ’BBB’. Make sure that the ’Philips’ head is visible.
(b) Align the small arrow visible on each cam lock so that it points outwards (in direction of the ’BB profile’).
(c) Slide the T-nuts (M3, 4 per ’BBB’) from one end into the corresponding ’BB profile’ (2 per profile). The required positioning is done in the next step.
(d) Loosely screw the M3×30 screws into the T-nuts (M3) and use them to align the nuts along the ’BB profiles’ according to the holes of the ’BBB’. Tighten the screws until they make contact with the profile and screw back a quarter turn.
(e) A perfect alignment is not yet required as the holes provide sufficient tolerance and the T-nuts (M3) can be moved easily.
(f) Optional: Add the desired amount of T-nuts (M4) for mounting components to the grooves of each ’BBB’.

### 5.3 Assembling a 3 x 3 optical breadboard

In this section the assembly of an optical breadboard is demonstrated. Exemplarily an optical breadboard size of 3 x 3 was chosen since it covers all necessary steps for any type of shape and size. The build-up of other sizes and/or shapes can be performed in a similar way.

(a) Prepare a total of nine (9) ’BBBs’ and four (4) ’BB profiles’ (36 cm long) to assemble a 3 x 3 breadboard (like in Section 5.2).
(b) There should be two (2) ’BB profiles’ (outer positions) with six (6) T-nuts (M3) and bolts (M3) on one side and two (2) ’BB profiles’ (inner positions) with a total of twelve (12) T-nuts (M3) and bolts (M3) on two opposing sides.
(c) Start from the outside using one of the outer ’BB-profiles’. Slide a ’BBB’ sideways on to two (2) M3×30 screws and insert the tongue of the ’BBB’ into the groove of the ’BB profile’ at the same time.
(d) Avoid over-tightening the cam locks to prevent any damage! To tighten the cam locks turn them clockwise with 3 Nm of torque.
(e) Repeat the previous step until three ’BBBs’ are connected to the ’BB profile’. Tip: As long as the cam locks are not fully tightened, the ’BBB’ can easily slide into position.
(f) Next, connect an inner ’BB profile’ to the ’BBBs’ and repeat the previous steps. To get the alignment of the T-nuts (M3) right a pencil can be used to roughly mark the required positions.
(g) Add another row of ’BBBs’ followed by an inner ’BB profile’ and finish the 3 x 3 breadboard with a final row of ’BBBs’ in combination with an outer ’BB profile’.

### 5.4 Design decisions and possible alternatives

The choice of industrial aluminum extrusions was deliberate in the realization of the optical breadboard. Reasoning behind this decision is the resulting stiffness which was carefully considered against the limited reusability as well as the low material price of aluminum, itself. It should be emphasized at this point that ’item Industrietechnik GmbH’ is only one possible manufacturer of aluminum extrusions. Alternatively, ’Bosch Rexroth AG’ and other manufacturers can be used, also. In this case, the feather of the ’BBB’ may then have to be slightly adjusted depending on the profiles’ shape. Although we cannot recommend this procedure, it is possible to use only single (12 cm long) ’BB profile’ with threaded holes at both ends. Theoretically, these ’BB profiles’ can then be connected to each other with a set screw, but practically the misalignment that occurs through twisting turns out to be a major issue.

An extraordinary aspect that has not yet been fully addressed is the incorporation of the existing product catalog from, e.g. ’item Industrietechnik GmbH’ or ’Bosch Rexroth AG’. While this work primarily focuses on aluminum extrusions in the context of an optical breadboard, it is also possible to use all further available design and construction tools of the respective companies. For example, necessary components to create a subframe for incorporating the optical breadboard in an industrial environment are directly available at the online store of ’item Industrietechnik GmbH’. There are no limits to the potential mechanical constructions.

To create a rigid foundation with a minimum of flexibility a honeycomb structure was directly embedded into the design of the ’breadboard brick’. An approximate density of 30% was chosen, which serves as a well trade-off between the stability and the amount of material required. For the specific dimensions a lower landwidth limit of 1.5 mm and at least 3.0 mm for the outer rims was chosen. By aiming for a periodical structure along the long axis we ended up with the specific dimensions shown in Fig. 1. Material wise there are two alternatives worth mentioning, PETG and ABS. The primary differences, apart from the printing properties, are a different elasticity and temperature resistance. Depending on the final application it might be advisable to choose the filament which suits the individual requirements best. In this work we chose PLA primarily because of its ease to print in a prototyping context and its outstanding damping features.

The reasoning behind the special T-nuts can be boiled down to two aspects. First, these nuts have a spring-loaded steel ball, which fixes them sufficiently in the groove but at the same time makes them easy to move and handle. Secondly, there are different threaded options (M3/M4/M5) for the same physical size of T-nut, which offers a great versatility for mounting components onto the ’breadboard brick’. A budget alternative is to use ordinary nuts but have in mind that this may require a modification of the grooves of the ’breadboard brick’. In addition, this type of fastening offers a long lifespan compared to threads cut directly into the ’breadboard bricks’. This way, repeated repositioning and fixing of optics is not a concern and reflects the intended case of use.

## 6 Operation instructions

Since the scalable optical breadboard presented in this work primarily serves as a platform for optical setups/experiments the instructions for operation is foremost dependent on the corresponding setup. Nevertheless, a few general operation instructions can be summarized in this chapter to provide the user with an optimal experience. In addition, laser safety issues must be addressed in the context of optical experiments made available to the general public.

### 6.1 General operation instructions

• It is considered good practice to plan the desired layout of the optical experiment beforehand to derive the required dimensions of the optical breadboard.
• While planning have in mind that it is generally faster to add a new row of ’breadboard bricks’ compared to swapping out the ’BB profiles’ for longer ones.
• For an easy alignment of the beam path it is advisable to direct the laser light along the inline geometry whenever possible.
• The modularity of the whole system allows the user to mount and pre-align related components, e.g. for building a telescope or a spatial frequency filter onto a subset of ’breadboard bricks’ even before the final assembly.
• Avoid over-tightening of bolts since the 3D-printed components are prone to deform or break especially when utilizing low infill levels. A maximum of 1.0 Nm of torque is advisable when mounting optics in the rails of the ’breadboard bricks’ (valid for PLA filament).
• The use of an enclosure for the optical breadboard is strongly recommended and can be easily realized by utilizing additional ’breadboard bricks’ or sufficient sheet material available from ’item Industrietechnik GmbH’.

### 6.2 Safety precautions

• If not smoothed, the sharp edges of the ’BB profiles’ pose a risk of injury.
• The usual safety regulations for working with laser light and electronics apply. In case of any uncertainty it is strongly recommended to look up the related safety regulations elsewhere.
• **Always handle laser light with care! Reflected and scattered light can easily damage or blind your eyes! It may also cause skin incineration and can light up flammable materials.**

## 7 Validation and characterization

In this chapter different representative geometric shapes are build and presented, to give a better impression of the possible designs and applications. Furthermore, the validation of the hardware with respect to its stiffness, high precision and damping is performed by means of the fringe stability of a Michelson–Morley interferometer.

### 7.1 Possible geometrical shapes

**Fig. 4:**
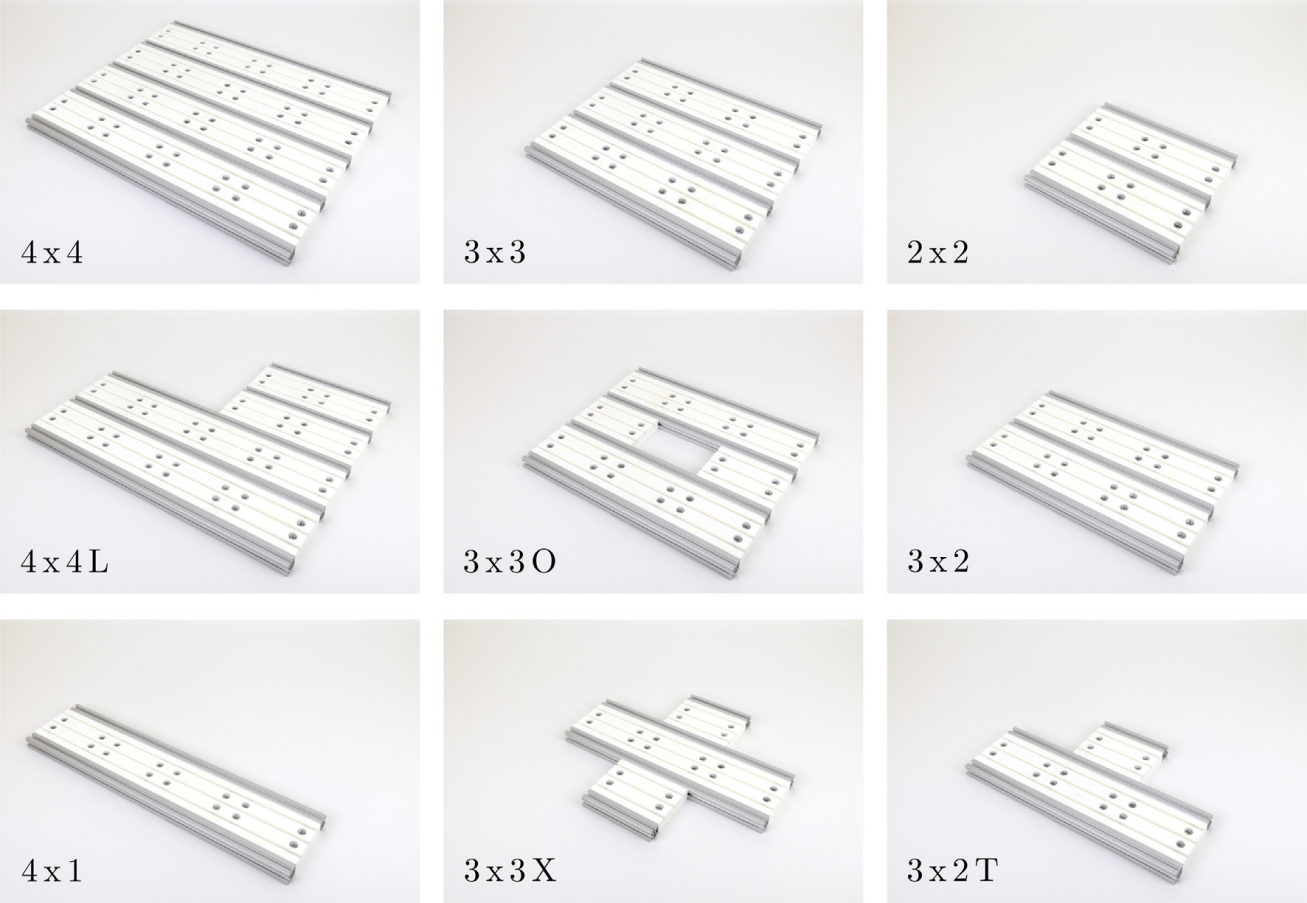
Showcase of possible geometrical shapes.

Fig. 4 shows different geometric shapes up to a size of 4 x 4 demonstrating the modularity and agility of the presented optical breadboard. Besides the basic rectangular shapes in different sizes (2 x 2, 3 x 3, 4 x 4), an L, O, U or X shape can easily be realized by utilizing only a few ’BB profiles’ of different lengths. The derived shapes are transferable to larger dimensions and can be freely combined with each other to create customized solutions. As simple example, a T shape is perfectly suited for coupling several laser sources into an experiment, whereby each source can be mounted in one arm of the T shape and switched between via a flip mirror. In one of its simplest forms, a 4 x 1 shape, its resemblance and functionality to the original ’Leybold GmbH’ optical bench is uncanny.

Key feature is the balance between the stiffness through incorporating continuous industrial aluminum extrusions and the agility achieved along one dimension through the chosen quick connecting mechanism. This agility results from the possibility to disassemble the entire system back into its components within minutes and to rebuild it into a new shape or, in the most simple case, to extend it.

### 7.2 Interferometer

**Fig. 5:**
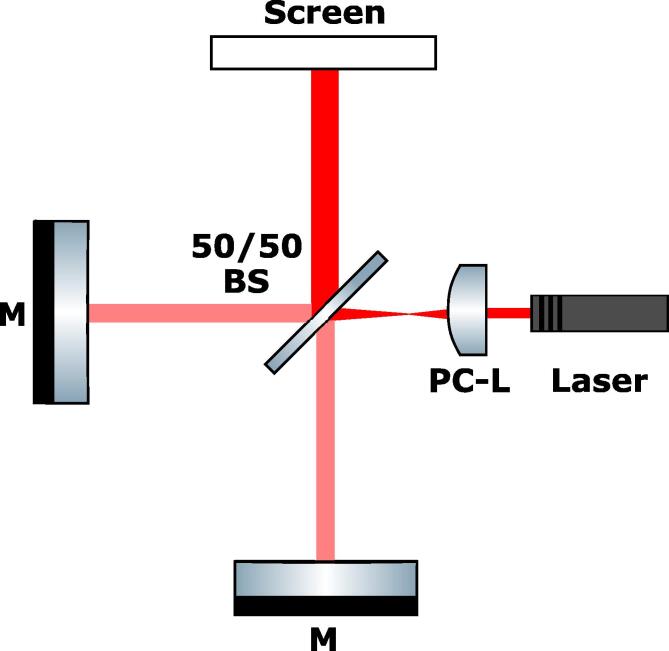
Sketch of the optical paths in a Michelson–Morley interferometer: The laser light is aligned through a plano-convex lens (PC-L) and is split using a 50/50 beamsplitter (50/50 BS). The resulting beams are reflected at two silver mirrors (M) along optical paths with a slight difference in the optical path lengths. The recombined beam is directed on a viewing screen.

We have build a Michelson–Morley interferometer on a 3 x 3 base to validate the stiffness and damping properties of our optical breadboard. Although the achieved stiffness is highly dependent on the used environment and the smallest vibrations may negatively impact the interference pattern, we chose a normal desk in one of our office rooms for the validation process to create a level playing field. Therefore, the shown results should be easily reproducible at home, e.g. in ones basement. A basic design was used for the layout, consisting of two protected silver mirrors (’Thorlabs GmbH’, type: PF10-03-P01), a 50/50 beamsplitter plate (’Thorlabs GmbH’, type: BSW10R), a plano-convex lens (’Thorlabs GmbH’, type: LA1608-ML, f = 75 mm) and a 650 nm laser diode (’Picotronic GmbH’, type: DB1650-1–3-FA-F3400, P ⩽ 1 mW) as depicted in Fig. 5.

**Fig. 6:**
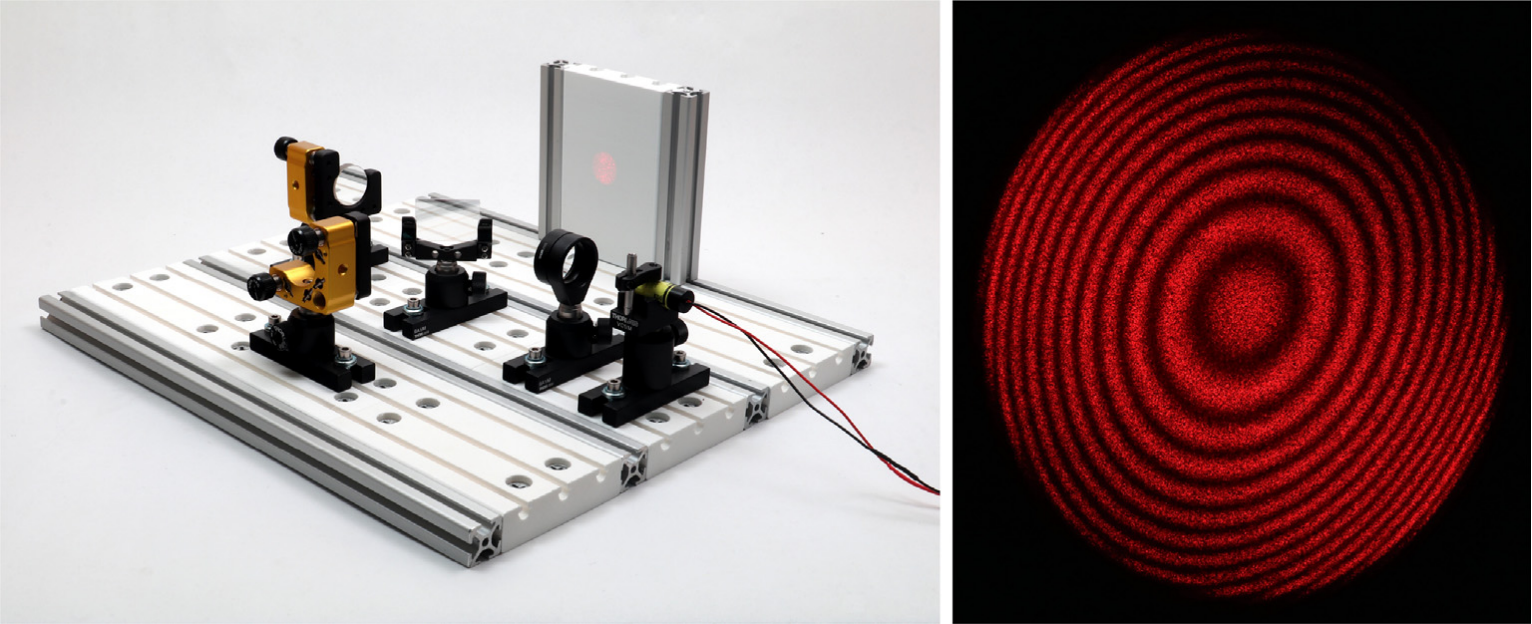
Left: Photograph of the completed Michelson–Morley interferometer build on a 3 x 3 breadboard. Right: Photograph of the resulting high contrast interference pattern indicating high stability of the fringe pattern as well as high precision of adjustment.

The optics are mounted onto the breadboard by mirror mounts from ’Liop-Tec GmbH’, type: SR100-HS-100L-2-YE, and the standard 1” system from ’Thorlabs GmbH’ consisting of a base plate (type: BA1), a 20 mm postholder (type: PH20) and a 20 mm post (type: TR20) were used. The final assembly and the accomplished interference pattern are shown in Fig. 6. The depicted concentric fringes can be interpreted as two curved wavefronts interfering with a small deviation in the relative path lengths of each arms. Noteworthy is the high contrast achieved, indicating great stability of the fringe pattern and sufficient precision of adjustment in the order of ≈0.2μm. Thereby, both, a good mechanical stiffness and good damping properties are validated.

**Fig. 7:**
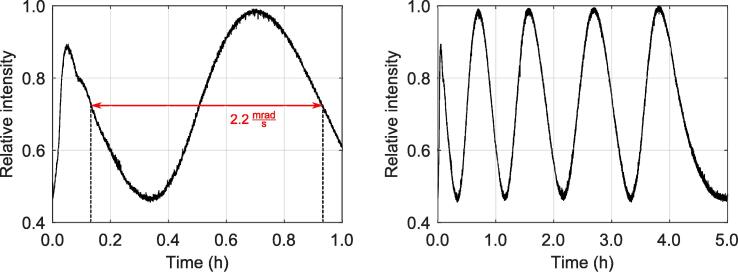
Relative intensity variation of the interference pattern’s center through a 1.5 mm pinhole as a function of time. The breadboard was placed onto a standard desk at ambient room temperature, ambient air pressure and in presence of common building vibrations. Left: The response immediately after adjustment. Right: The intensity variation over a period of five hours.

Fig. 7 further provides the relative intensity variation of the interference pattern’s center measured through a 1.5 mm pinhole as a function of time over a period of five hours. For this measurement, we highlight, that the breadboard was placed onto a standard desk in one of our offices, i.e. within an environmental condition of ambient temperature, ambient air pressure and in presence of common building vibrations (2nd floor). Furthermore, a standard, not-temperature-stabilized, battery-driven laser system at a wavelength of λ=650nm was used. Data acquisition with a commercial laser power meter (’Coherent Inc.’, type: LabMax-TOP  + OP-2 VIS) started immediately after final adjustment of the interferometer. We assume that these conditions best correspond to the anticipated field of application of the presented breadboard: agile prototyping in the field of optics & photonics. As a result, already after a few minutes a quasi-stable state was reached (Fig. 7, left), that is characterized by a phase shift over time of 2.2 mrad/s, corresponding to λ/50 per minute. In this time range, the amplitude is determined with an RMS noise amplitude of about 5%, that is in the limit of the detector’s noise of the power meter. On the long term, the phase stability has further improved (Fig. 7, right) validating the high quality of this breadboard.

## Declaration of Competing Interest

The authors declare that they have no known competing financial interests or personal relationships that could have appeared to influence the work reported in this paper.
